# The Riddle of Cetuximab-Related Skin Toxicity: ^1^H-NMR Sebum Analysis Revealed Dynamic Lipid Alterations Associated with Skin Toxicity Development in Metastatic Colorectal Cancer Patients

**DOI:** 10.3390/cancers14215308

**Published:** 2022-10-28

**Authors:** Asia Saorin, Emanuela Di Gregorio, Angela Buonadonna, Gianmaria Miolo, Giuseppe Corona

**Affiliations:** 1 Immunopathology and Cancer Biomarkers Unit, Centro di Riferimento Oncologico di Aviano (CRO), IRCCS, 33081 Aviano, Italy; 2 Department of Molecular Sciences and Nanosystems, Ca’Foscari University of Venice, 30170 Venice, Italy; 3 Medical Oncology and Cancer Prevention Unit, Centro di Riferimento Oncologico di Aviano (CRO), IRCCS, 33081 Aviano, Italy

**Keywords:** cetuximab, cancer, sebum, ^1^H-NMR, skin, toxicity, lipidomics

## Abstract

**Simple Summary:**

The etiology of the skin toxicity that is a common side effect of cetuximab is still under debate. In this study, proton nuclear magnetic resonance (^1^H-NMR) analysis of the sebum of 30 metastatic colorectal cancer patients, collected at different time points during cetuximab administration, was performed. Both sebum production and composition were found to be affected by cetuximab treatment. These specific sebum lipid alterations could help to better understand the development of cetuximab-induced skin toxicity and could be useful for improving skin lesion prevention and treatments.

**Abstract:**

The epidermal growth factor receptor inhibitor (EGFRIs) treatments are commonly associated with the development of adverse skin effects. This study aims to investigate the lipid composition change in sebum during cetuximab-based treatment in an attempt to identify specific metabolic signatures useful in predicting the occurrence of severe skin toxicity. Sebum from 30 metastatic colorectal cancer (mCRC) patients was collected at three time points during the targeted therapy by the application of Sebutape^®^ on the forehead, and the major lipid classes were analyzed and quantified by ^1^H-NMR. Univariate analysis was performed to reveal significant alterations among patients in sebum production as well as lipid composition and over the course of cetuximab therapy. A transient but significant decrease in sebum production associated with a reduction in the relative content of triglycerides (TG) and squalene (SQ) was found to be induced by cetuximab administration. The reduction of these two lipid classes was also found to be associated with the severity of skin rash experienced by patients. The results of this study indicate that cetuximab-based treatment can reduce sebum gland activity, leading to an overall decrease in sebum production and the induction of specific modifications to its composition. The extent of the loss of skin barrier function may be important for determining the severity of skin toxicity development.

## 1. Introduction

Epidermal growth factor receptor (EGFR) is a tyrosine kinase transmembrane protein expressed in healthy tissue such as the gastrointestinal tract, epithelial tissues, skin, and hair follicles, and it is also involved in tumor proliferation. Given its role in cancer progression, several EGFR inhibitors (EGFRIs) have been developed in the form of monoclonal antibodies, namely cetuximab (Erbitux^®^) and panitumumab (Vectibix^®^), as well as in the form of small-molecule drugs such as erlotinib (Tarceva^®^) and gefitinib (Iressa^®^). Compared with the standard cytotoxic effects of conventional chemotherapeutic drugs, these target EGFR therapies are characterized by low systemic side effects [1]. However, specific local EGFRIs side effects are severe enough to reduce patients’ quality of life and require dose reduction or the discontinuation of treatment, as in the case of skin toxicity [2]. A dose-dependent follicular papulopustular eruption, also known as acneiform rash, represents the most common side effect, mainly localized on the chest, scalp, face, and upper back. It usually appears within 1 or 2 weeks from the beginning of the therapy, and it is characterized by a high incidence, reaching 80–90% in the case of metastatic colorectal cancer (mCRC) patients treated with EGFRIs [3,4,5].

Although the pathogenesis of such skin toxicity is not fully understood, it is known that EGFR plays a pivotal role in the epidermis development since keratinocyte differentiation and migration toward the upper layer of the epidermis is dependent on EGFR signaling [6]. Indeed, the inhibition of EGFR in cancer patients leads to skin toxicity, whose severity has shown to be positively correlated with patients’ survival and hence could be a surrogate marker of tumor response to EGFRIs [7]. Understanding the causes of skin toxicity and revealing biomarkers capable of predicting it could help to improve patient management of skin toxicity as well as to predict the outcome of cetuximab-based therapies.

This study aims to investigate by ^1^H-NMR technique (which allows a straightforward quantification of different classes of lipids simultaneously) the potential sebum lipid alterations in RAS wild-type mCRC patients undergoing cetuximab treatment [8]. In particular, the study focuses on the analysis of sebum lipid alterations associated with skin toxicity during cetuximab administration in attempt to indicate the origin of its development and to predict its occurrence.

## 2. Materials and Methods

### 2.1. Characteristics of Patients

In this investigation, a group of 30 RAS wild-type mCRC patients were enrolled. The clinical characteristics of the patients, stratified according to the development of skin toxicity and chemotherapy type, are reported in Table 1.

Patients were subjected to different chemotherapy regimens constituted by CAPIRI, FOLFIRI, and FOLFOX, all associated with cetuximab administered at the dose of 500 mg/m^2^ biweekly. Patient rash was classified during go-rounds of chemotherapy according to Common Terminology Criteria for Adverse Events (CTCAE v3.0) [9]. The severity ranged from grade 1 to 4. Grade 1 involves papules and/or pustules covering less than 10% of the body that do not require any treatments; grade 2 requires the administration of topical antibiotics, while from grade 3, patients received a specific 100 mg oral tetracycline antibiotic treatment and required a temporary or definitive interruption of EGFRI treatment. The study was conducted in accordance with the Declaration of Helsinki and all patients gave written informed consent.

### 2.2. Sebum Sampling

Sebum samples were collected at different times during the progress of the chemotherapy, namely before the first administration (time point “a”, baseline) and after 14 and 28 days (time point “b” and “c”) from the start of the treatment, which correspond to the end of the first and second cycles of the therapy, respectively.

Sebum samples were collected by the application of a lipid-adsorbing tape (Sebutape^®^, Cuderm Corporation, Dallas, TX, USA) [10] according to the following procedure: the tape was removed from its holder and placed on the forehead of the subjects, previously cleaned by 70% ethanol/water solution to remove the lipid skin layer. After 30 min of time collection, the tape was removed, folded in half, placed in 1.5 mL Eppendorf^®^ (Eppendorf, Hamburg, Germany) tube and then stored at −80 °C. All of the procedure was performed using tweezers.

Sebum samples from a healthy volunteer were used to establish sebum analysis ^1^H-NMR method. Clean, blank tapes were subjected to the same extraction procedure and were used to evaluate the presence of interfering signals resulting from the polymeric matrix of the tape.

### 2.3. Extraction of Sebum Lipids

Prior to lipid extraction from the Sebutape^®^ the non-adsorbing black tabs of tape were cut-off and its remainder placed into a 1.5 mL Eppendorf^®^ tube containing 1 mL of isopropanol (PanReac AppliChem, 131090.1211, Barcellona, Spain). Different solvents such as chloroform, ethanol, and cyclohexane were previously reported to efficiently extract lipids from Sebutape^®^ [8,11,12,13]. However, isopropanol allowed us to obtain a better signal-to-noise ratio in the ^1^H-NMR measurement. After solvent addition, the tape was vortexed for 10 s three times, bath sonicated for 20 min (cooled by ice), then vortexed again for 10 s three times. After, the solvent was transferred into a clean 1.5 mL Eppendorf^®^ tube and centrifuged at 13,000× *g* rpm for 5 min to eliminate traces of skin/hairs present in the extract. A volume of 800 µL of the supernatant was placed into a clean Eppendorf^®^ tube and evaporated under vacuum. The dried extract was then resuspended in 600 µL of deuterated chloroform (Merck, 151823, Darmstadt, Germany) by 10 s of vortex followed by 1 min of ultrasonic bath and placed in the NMR tube.

### 2.4. ^1^H-NMR Analysis of Sebum Lipids

Sebum lipid extracts were subjected to ^1^H-NMR analysis to quantify the total amount of sebum as well as to characterize their lipid composition. Data were acquired by Bruker 400 MHz Advance spectrometer (Bruker, Billerica, MA, USA). Acquisition parameters consisted in a 20.02 ppm spectral width, 30° pulse, 4.08 s acquisition time, 1 s relaxation delay, 64,000 data points, 1600 scans. MestReNova^®^ (Mestrelab Research, Santiago de Compostela, Spain) software was used for processing proton free induction decay data typically by Fourier transformation, and phasing and spectra were referenced to the low levels of protonated chloroform. Only signals with relative standard deviations and relative interference intensities lower than 10% were considered. The sum of sebum peak areas was linearly correlated (R^2^ = 0.995) to the sebum amount measured by differential weighing of Sebutape^®^ (before and after the application). The ^1^H-NMR lipid signals were assigned to sebum components using standard lipids which are representative of the main lipid classes present in sebum (Figure S1). The annotated signals were baseline corrected and peak integration values were normalized using matrix reference peak (5.0 ppm). Lipid concentrations were calculated from external standard calibration curves obtained with standard compounds.

### 2.5. Statistics

The data are reported as average ± standard deviation (SD), or standard error of the mean (SEM), or in the case of a non-normal distribution (established by Shapiro test), by median and interquartile ranges (IQR). Comparisons are performed by *t*-test (paired or unpaired) or with non-parametric Wilcoxon or sign tests. The differences among the investigated groups were considered significant for *p* < 0.05. Analysis and graphing are performed with RStudio software (RStudio Team (2021), version 4.1.2 (2021-11-01), Boston, MA, USA).

## 3. Results

### 3.1. ^1^H-NMR Analysis of Sebum Lipids

^1^H-NMR sebum lipid analysis, compared with high performance liquid chromatography (HPLC), which is also applied for sebum analysis [14], does not require complex sample preparation or the addition of standards for each lipid class. Therefore, ^1^H-NMR allows us to obtain quantitative data simply by the integration of peak areas [8].

**Figure 1:**
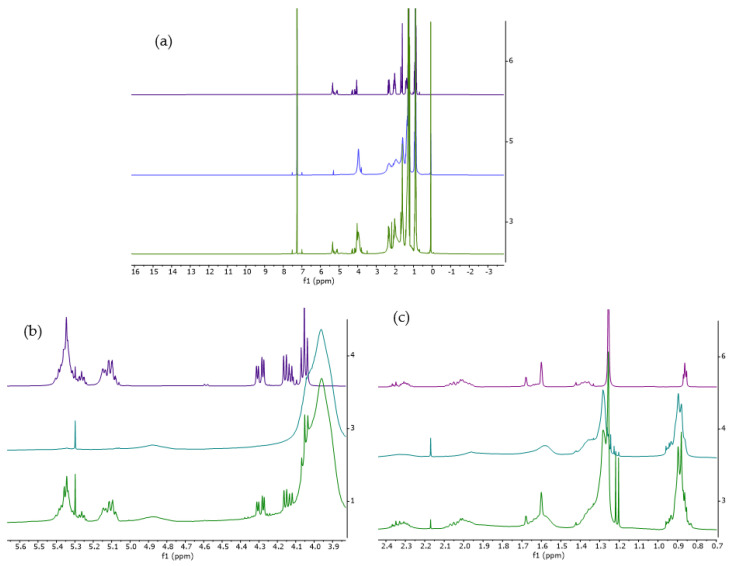
Spectra of an applied tape (green), a clean tape (blue), and in violet, extracted peaks assigned to sebum. (**a**) Total spectra, (**b**) magnification of double bond HC = C and ester region, and (**c**) CH and CH2 deshielded and CH2, CH3 regions.

Figure 1 displays representative ^1^H-NMR sebum spectra, while signal assignments to lipid classes are reported in Table S1. There is no evidence of lipid oxidation products in high ppm regions (5.1 to 10 ppm) [15]. Moving to lower ppm, a certain contribution of the matrix is present, especially for CH2, CH3 regions (1.4 to 0 ppm).

### 3.2. Sebum Production

Patients treated with cetuximab commonly manifest dry itchy skin, and hence monitoring sebum production during the treatment may be of interest. Sebutape^®^ allows the efficient collection of the sebum secreted by sebaceous glands. In order to establish the optimal time required to collect the maximum amount of sebum, we quantified the sebum of a healthy volunteer obtained from tapes removed at different application times. In about 20 min the amount of sebum becomes constant (Figure S2), indicating that a sampling time of 30 min is adequate to collect the maximum amount of sebum. The average amount of forehead sebum sampled before the start of the therapy (time point “a”) is equal to 1.0 ± 0.5 mg, in agreement with a 150–300 μg/cm^2^ normal distribution [16].

### 3.3. Sebum Lipid Composition

**Figure 2:**
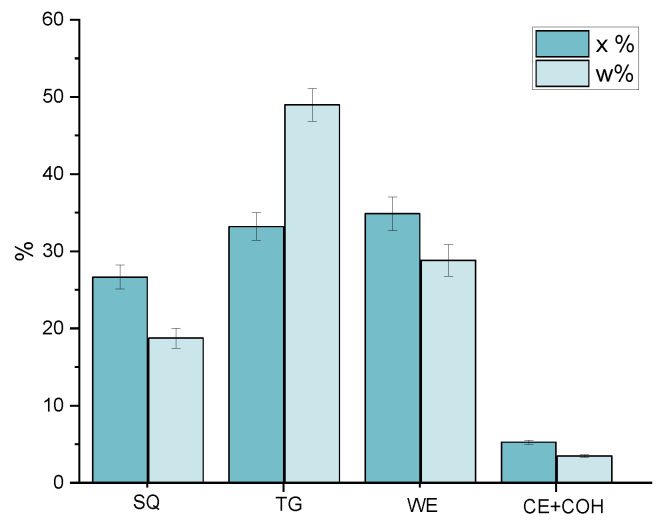
Quantified lipid mole fraction (x%) and weight (w%) percentages determined in sebum samples collected from patients before the start of the therapy. Data are reported as average ± SEM. SQ: squalene, TG: triglycerides, WE: wax esters, CE: cholesterol esters, COH: cholesterol.

The determined average ^1^H-NMR lipid sebum composition is in agreement with that previously reported [8,16]. Indeed, the quantitative ^1^H-NMR measurements pointed out that triglycerides (TG) represent the main sebum lipid component (49 ± 2 w%), followed by wax esters (WE) (29 ± 2 w%) and squalene (SQ) (19 ± 1 w%), while cholesterol (COH) and cholesterol esters (CE) are minor components (3.5 ± 0.2 w%) (Figure 2).

### 3.4. Effect of Cetuximab Treatment on Sebum Production

**Figure 3:**
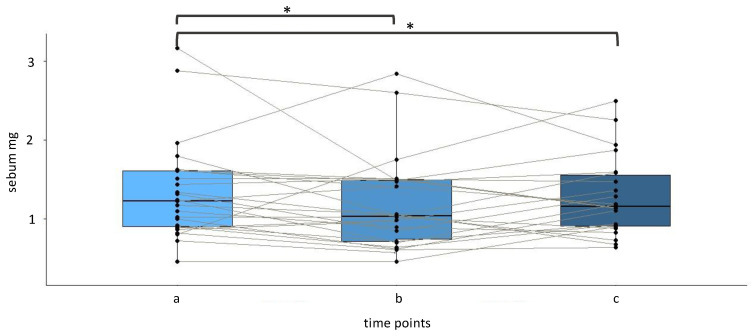
Effect of cetuximab-based therapy on the overall sebum production evaluated at baseline (“a”) and after 14 and 28 days of treatment (“b” and “c”); * *p* < 0.05 by sign test.

The total sebum amount was determined at the three time points “a” (baseline), “b” and “c” (along therapy). ^1^H-NMR data revealed a statistically significant decrease in sebum production after 14 (b: 0.8 ± 0.5 mg) and 28 (c: 0.9 ± 0.4 mg) days of treatment when compared with the baseline level (a: 1.0 ± 0.5 mg) (Figure 3).

### 3.5. Effect of Cetuximab Treatment on Sebum Lipid Composition

**Figure 4:**
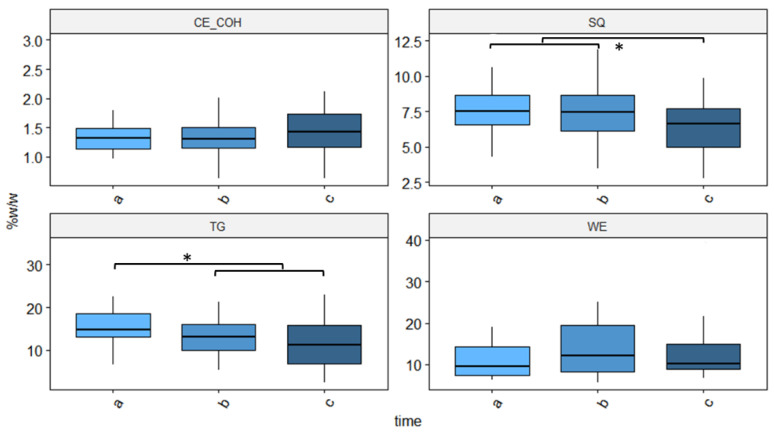
Change in sebum lipid composition induced by cetuximab treatment. Lipid concentrations are expressed as weight percentage of total sebum (%, *w*/*w*). The significance of time point (baseline “a”, 14 and 28 days after the start of the treatment respectively “b” and “c”) differences was evaluated by paired *t*-test and Wilcoxon test; where *p* is lower than 0.05, * are reported.

The comparison of sebum composition expressed as weight percentage of lipids on total amount of sebum (%*w*/*w*) among the three investigated time points is reported in Figure 4. This analysis revealed that SQ decreased from 7.6 ± 0.4% and 7.3 ± 0.4% at time points “a” and “b”, respectively, to 6.2 ± 0.4% at time point “c”. A significant decrease was also determined for TG, which showed a basal value of 15 ± 1% that dropped to 13 ± 1% and 12 ± 1% at time points “b” and “c”, respectively.

### 3.6. Skin Rash Grade as Function of the Different Chemotherapy Regimens

Patients were subjected to different chemotherapeutic regimens in association with cetuximab administration, classified as irinotecan- (FOLFIRI, CAPIRI) or oxaliplatin (FOLFOX)-based chemotherapy. In order to evaluate if the severity of the rash is influenced by different kinds of chemotherapy, we stratified patients according to the above chemotherapy regimens. Performing Fisher’s exact test for non-significant differences revealed the development of severe skin rash toxicity (Table 2).

### 3.7. Skin Rash Grade as Function of Patients’ Sex and Age

**Figure 5:**
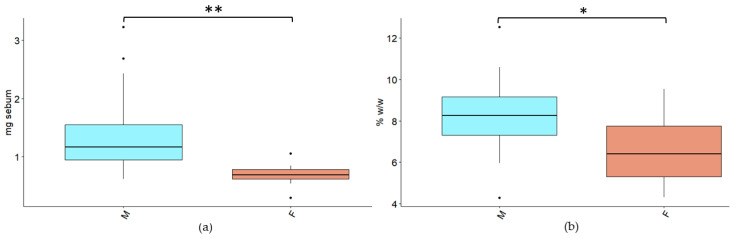
Differences in sebum amount and composition related to patients’ sex at baseline. (**a**) Sebum amount expressed in mg and (**b**) squalene percentage (%*w*/*w*). *t*-test was performed, where *p* is lower than 0.05 and 0.01, * and ** are respectively reported.

Sebocyte activity is affected by hormones both in terms of production and composition [17], suggesting possible differences between male and female groups in the development of skin rash toxicity. For this reason, even if the number of patients is limited and not equally balanced in terms of sex, age, and other factors, for explorative purposes patients were subdivided into subgroups in order to evaluate the influence of other phenotypic characteristics in the development of skin rash. The present study confirms a different level of sebum production and specific lipid composition between male and female patients. At baseline the male group showed a significantly higher production of sebum with a higher percentage of SQ compared with the female group (Figure 5).

**Figure 6:**
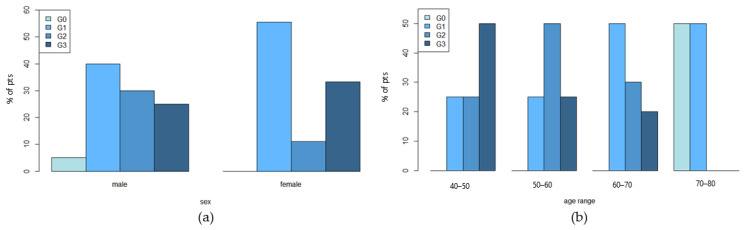
Percentage of patients experiencing different rash grade grouped by sex and age. (**a**) Percentage of patients in each rash group clustered by sex and (**b**) male patients grouped by age.

In spite of these differences, male and female patients reported non-significant differences in terms of severity of rash grade. However, male patients stratified by age showed a decreasing trend in rash grade associated with increasing age but without reaching statistical significance by Fisher’s exact test (Figure 6). This trend was not observed in the female group since it was likely comprised of a lower number of patients.

### 3.8. Skin Rash Grade and Sebum Alterations

**Figure 7:**
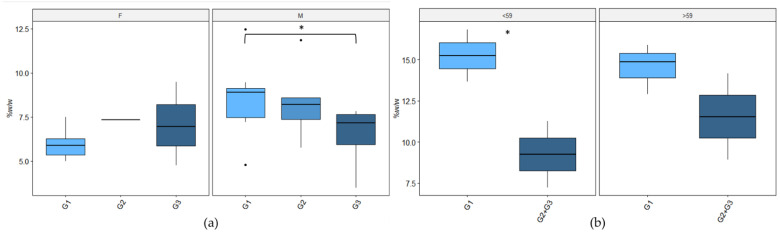
Sebum alteration associated with different grades of rash experienced by patients grouped by sex and age. (**a**) Male and female patient variations in SQ percentage at time point ”b” (the G0 patients were excluded). (**b**) Level of TG at time points “a” for female patients grouped by their age, considering 59 as cut-off age. Wilcoxon test was performed, where *p* is lower than 0.05, * are reported.

Sebum variation associated with rash severity is of particular interest since it could be relevant for the identification of predictive biomarkers. In this context, patients were stratified according to rash grade, and alterations in sebum production and composition were evaluated, although without indicating any significant lipid pattern that can be associated with skin toxicity. Conversely, when the patients were stratified by sex, some interesting differences emerged. For male patients, a decreasing trend of SQ percentage at time point “b” was associated with the increase in rash grade (9 ± 1% for G1, 8 ± 1% for G2, and 7 ± 1% for G3), while in the female group no differences were highlighted. Given that hormones can influence sebum production and composition and differ based on menopausal status [18], female patients were further divided according to their age, considering the age of 59 years as a cut-off. The TG percentage at time point “a” resulted as higher for younger women experiencing lower skin side effects, and the same trend was present for older patients even if it did not reach statistical significance (Figure 7).

## 4. Discussion

In this study, we applied the ^1^H-NMR technique to determine information on sebum production and lipid composition in a group of mCRC patients subjected to cetuximab treatment. Compared with other principal analytical techniques applied in metabolomics, such as gas and liquid chromatography coupled with mass spectrometry, ^1^H-NMR allows an easier sample preparation and the direct absolute quantification of metabolites with a higher reproducibility. The use of Sebutape^®^ represents an interesting breakthrough in sebum analysis since this specific matrix device, composed by a hydrophobic, open-celled, microporous polymeric film coated with an adhesive layer [10], allows us to collected sebum in a time-dependent manner. The application of the tape for 30 min was sufficient to obtain quali–quantitative ^1^H-NMR spectra from a single tape, without requiring the use of a cryoprobe device to improve sensitivity. The ^1^H-NMR analysis of the sebum extracts showed specific previously reported matrix signals corresponding to components of the adhesive layer of Sebutape^®^ [12,14,19]. However, matrix signals were not found to interfere significantly in the quantification of lipids. On the other hand, such signals were instead found useful for normalization, allowing us to eliminate the variation deriving from sample preparation without the need of adding an internal standard and to correct for the different tape‘s areas subjected to extraction.

In patients, the mean amount of sebum after 30 min of collection estimated by ^1^H-NMR measurement was found in agreement with that reported in a previous study [16]. Since the forehead is a rich sebaceous gland area, it is likely that lipid components have such preferential origin, with a negligible contribution deriving from keratinocytes [16]. The main component of the secreted sebaceous lipids was represented by TG, which constitute the 45% of the sebum weight, followed by WE (25%), SQ (12%), free fatty acids (FA) (10%), COH, and CE (4%) and diglycerides (DG) (2%). TG and WE have signals that can be assigned to all the individual lipids belonging to these two classes. Indeed, the α proton of esters and the methylene proton of glycerol show, respectively, a triplet and a doublet that are not shifted by different alkyl chains. Accordingly, COH and CE were identified by a methyl proton in the cholesterol structure common for all the lipids of this class. Conversely, SQ is the only lipid that can be individually quantified, while the characteristic triplet of the methylene proton of FA is part of a multiplet which cannot be distinguished (Table S1).

Sebum production’s quantification is of particular interest since one of the common skin issues manifested by cetuximab-based treatments consists in itchy dry skin [20]. In this context, sebum production during cetuximab treatment was observed significantly decreased after 14 (first cycle, “b”) and 28 days of treatment (second cycle, “c”), indicating that cetuximab treatment may induce a drop in the sebum production which is not completely restored on the second therapeutic cycle.

In contrast with this observation, a previous study analysed the sebum variation of eight non–small-cell lung cancer patients treated with either gefitinib or erlotinib [21] and reported instead an increased level of sebum in patients experiencing acneiform rash [21]. Conversely, sebum amount alteration, evaluated in 15 patients with head and neck cancer treated with cetuximab or panitumumab, showed a reduction in the symptomatic patients [22], in agreement with the present investigation. Such a discrepancy can be linked to the observation that acneiform rash is most frequently triggered by monoclonal antibodies compared with the small-molecule tyrosine kinase inhibitors [3]. Therefore, the different toxicity could be explained as different effects of drugs on sebaceous cells that consequently affect sebum production. Interestingly, the skin toxicity observed was not influenced by the kind of chemotherapy associated with cetuximab (Table 2), suggesting that it is prevalently dependent on cetuximab administration. In this context, the sebum production drop experienced by patients during the treatment could be essentially associated with the abnormal keratinocyte differentiation determined by cetuximab, which in turn affects sebaceous gland functions [23]. This specific inhibitory effect has been confirmed on skin lesion biopsies of patients subjected to high doses of EGRIs, where less ordered sebaceous gland differentiation and mild distortion of the basal layer were observed [24]. It is worthy to note that defective sebum synthesis has also been reported to be related to skin inflammatory diseases such as atopic dermatitis and psoriasis [25]. Sebum is essential for skin health, being a barrier responsible for maintaining water content and homeostasis of skin microbiota, thus controlling both inflammation and oxidation [25,26]. Interestingly, these sebum functions are deeply related to EGFR, since skin immune homeostasis is EGFR-signal-dependent [27], and inflammation is an important factor involved in the development of EGFRIs skin reactions [3,23,28].

The present study revealed a significant specific decrease in the overall sebum levels of TG and SQ after 14 and 28 days (“a” and “b” time points, respectively) of cetuximab treatment. Such a decrease, from the start of treatment, can be trigged by the proliferation or the activation of Cutibacterium bacteria (formerly Propionibacterium). This latter is a lipolytic microorganism which transforms TG in glycerol and produces short chain FA, such as butyric and propionic acids, which are responsible for inducing inflammation [29]. The connection between EGFRIs and Cutibacterium has been defined since their combination is able to induce keratinocyte IL-36γ expression and drive IL-8-mediated neutrophil-rich inflammation [28]. Acne has also been associated with an abnormal Cutibacterium proliferation [30] and, although acne and EGFRIs rash were considered different processes in the past [3], similarities between the two skin issues recently seem to emerge. Indeed, both are characterized by folliculitis with massive infiltration of neutrophils, and they share the same sebum-rich body areas colonized by Cutibacterium.

In addition to the drop in TG, Cutibacterium can also be responsible for the SQ decrease with cetuximab treatment. The latter is a COH precursor that in humans only accumulates in sebum, having both lubricant and antioxidant properties, mainly associated with its numerous double bonds that can act as scavengers of reactive oxygen species (ROS) [31]. The SQ oxidation can be a consequence of porphyrins produced by Cutibacterium since porphyrins act as efficient photocatalysts for the generation of various forms of ROS [32]. Moreover, SQ peroxides have been reported to be involved in the production of inflammatory mediators [33], and high levels of these molecules were found in the comedones of acneic subjects [34].

In sum, the sebum decrease due to the sebaceous gland distortion induced by cetuximab treatment determines the loss of skin barrier function, which could induce skin dysbiosis characterized by an over development of Cutibacterium, which influences sebum composition and triggers skin inflammation.

Sebum amount and composition variations have been found to be associated with the differences in the development of cetuximab skin severity side effects. Stratifying patients according to sex revealed a decreasing trend of SQ associated with an increase in skin toxicity in male patients already after 14 days from the start of cetuximab administration. This alteration could suggest that in patients experiencing more intense rash, SQ consumption could start earlier. In the female group, stratified by age, the baseline levels of sebum TG were found to be significantly lower for G2–G3 skin rash (Figure 7), suggesting that higher pre-treatment levels of TG could help to better maintain skin barrier function and hydration [35]. The differences in sebum production and composition between males and females may be ascribed to their different hormonal status [17], further conditioned by menopause [18] (Figure 5). Conversely, only for the male group, age seems to be a possible preventing factor for severe skin-rash toxicity (Figure 6), likely due to the sebaceous gland shrinking that occurs with age [36].

Despite the application of ^1^H-NMR for the sebum lipids analysis being appropriate, proficient, and effective, the translation into the clinical setting could represent a significant cost increase to consider [37]. Conversely, the application of Sebutape^®^ for the monitoring of sebum production does not determine the introduction of extra costs.

## 5. Conclusions

The developed ^1^H-NMR method allowed the straightforward analysis of sebum, revealing a decrease in its production and in lipid composition induced by cetuximab treatment. The two main sebum components, TG and SQ, significantly decreased, respectively, after 2 and 4 weeks of cetuximab administration. These alterations could be explained assuming an abnormal colonization of Cutibacterium, indicated as a pivotal contributor in cetuximab skin toxicity development. Gender and age resulted in important factors in determining the sebum production and composition that in turn influence skin rash severity. Further analysis concerning the quantification of single-lipid species instead of the entire class and the inclusion of fatty acids profiles could prove useful to better understand the etiology of skin rash from cetuximab toxicity. It has been demonstrated that sex and other phenotypic characteristics can influence sebum production, composition, and also treatment response. Moreover, other characteristics not considered in this study, such as diet, body mass index, and medications, may also influence the occurrence of skin toxicity. Given the limited number of patients involved in the study, a large cohort of patients is required in order to properly evaluate other contributing factors. This could help to better establish the potential of baseline sebum ^1^H-NMR lipid profile as a predictive biomarker of the development of skin rash associated with cetuximab treatments. Despite its limits, this exploratory study revealed a possible connection between the underlying mechanisms of the cetuximab skin rash occurrence and acne development, thus giving the opportunity to translate acne treatment in the management of skin rash induced by cetuximab treatment.

## Figures and Tables

**Table 1:** Patient characteristics.

Characteristics	*n* (%)
Mean age (range)	60 (43–77)
Sex	
female	10 (33%)
male	20 (67%)
Chemotherapy	
FOLFIRI	20 (67%)
CAPIRI	3 (10%)
FOLFOX	7 (23%)
Skin rash grade	
G0	1 (3%)
G1	14 (47%)
G2	7 (23%)
G3	8 (27%)

**Table 2:** Skin rash toxicity as function of chemotherapy and sex.

	M		F
	irinotecan-based		
G0	0		0
G1	6 (40%)		5 (63%)
G2	5 (33%)		1 (12%)
G3	4 (27%)		2 (25%)
	oxaliplatin-based		
G0	1 (20%)		0
G1	2 (40%)		1 (50%)
G2	1 (20%)		0
G3	1 (20%)		1 (50%)

## Data Availability

The data presented in this study are available on request from the corresponding author. The data are not publicly available due to privacy.

## Supplementary Materials

The following supporting information can be downloaded at: https://www.mdpi.com/article/10.3390/cancers14215308/s1, Table S1: NMR: Selected sebum signal assignation; Figure S1: standard lipid molecules with numbering referring to proton assignment; Figure S2: ^1^H-NMR determined weight of sebum obtained at different Sebutape^®^ application times collected from healthy volunteer.
