# Supplementary material for: The Riddle of Cetuximab-Related Skin Toxicity: 1H-NMR Sebum Analysis Revealed Dynamic Lipid Alterations Associated with Skin Toxicity Development in Metastatic Colorectal Cancer Patients
Source: Cancers (Basel). 2022 Oct 28;14(21):5308. doi: 10.3390/cancers14215308 (PMC9656910; doi:10.3390/cancers14215308)
Supplement: Supplementary file 1 [file cancers-14-05308-s001.zip › cancers-1954216-supplementary.pdf]

Supplementary Materials: The Riddle of Cetuximab Related Skin Toxicity: ^{1}H-NMR Sebum Analysis Revealed in Metastatic Colorectal Cancer Patients Dynamic Lipids Alterations Associ-ated with Skin Toxicity Development

Asia Saorin, Emanuela Di Gregorio, Angela Buonadonna, Gianmaria Miolo and Giuseppe Corona

Table S1. NMR Selected sebum signal assignation.

Peak name	chemical shift (ppm)		multiplicity	type of protons	assignment
A	5.22	5.45	m	-C**H**=C**H**-, RCOH2-C**H**(OR')-CH2R''	unsaturated acyl groups and FA (e.g. TG(9,10,32,33,52,53), CE/COH(6), CE(39,38,40,41)), glyc-eryl group TG(21)
B	5.065	5.22	m	-C**H**=C	SQ (3,7,11,14,18,22)
C	4.57	4.61	m	RCOOC**H**	CE (3)
D	4.26	4.32	dd,dd*	ROC**H**2-CH(OR')-C**H**2OR''	TG (20,22)
E	4.175	4.115			
F	4.14	4.2	t	-C**H**2COOR	WE (15)
G	2.24	2.4	m	RCOOC**H**2- ,-C**H**2COOR	FA(2),DG(17,24), TG(17,25,44), WE(19),CE(30), COH/CE(4)
H	2.097	1.952	m	C**H**2-CH=CH	SQ(4,5,8,9,12,13,16,17,20,21), CE/COH(7,12) and unsatu-rated acyl chain and FA
I	1.67	1.68	s	-CH3	SQ (25,24)
L	1.61	1.58	s	-CH3	SQ (1,26,27,28,29,30)
M	1.24	1.26	s	-(CH2)n-	overlapping of methylene hydrogens in acyl groups
N	0.86	0.85	t	-CH3	
O	0.668	0.682	s	-CH3	CE,COH (18)

*other lower signals in this interval of ppm can be attributed to glycerol group of 1,3 and 1,2 DG.

Peaks B,D,F,O were selected as diagnostic ones and used for the quantification of squalene, pool of triglycerides, pool of wax esters and cholesterol/cholesterol esters, respectively. In the case of free fatty acids and diglycerides there are no selective peaks.

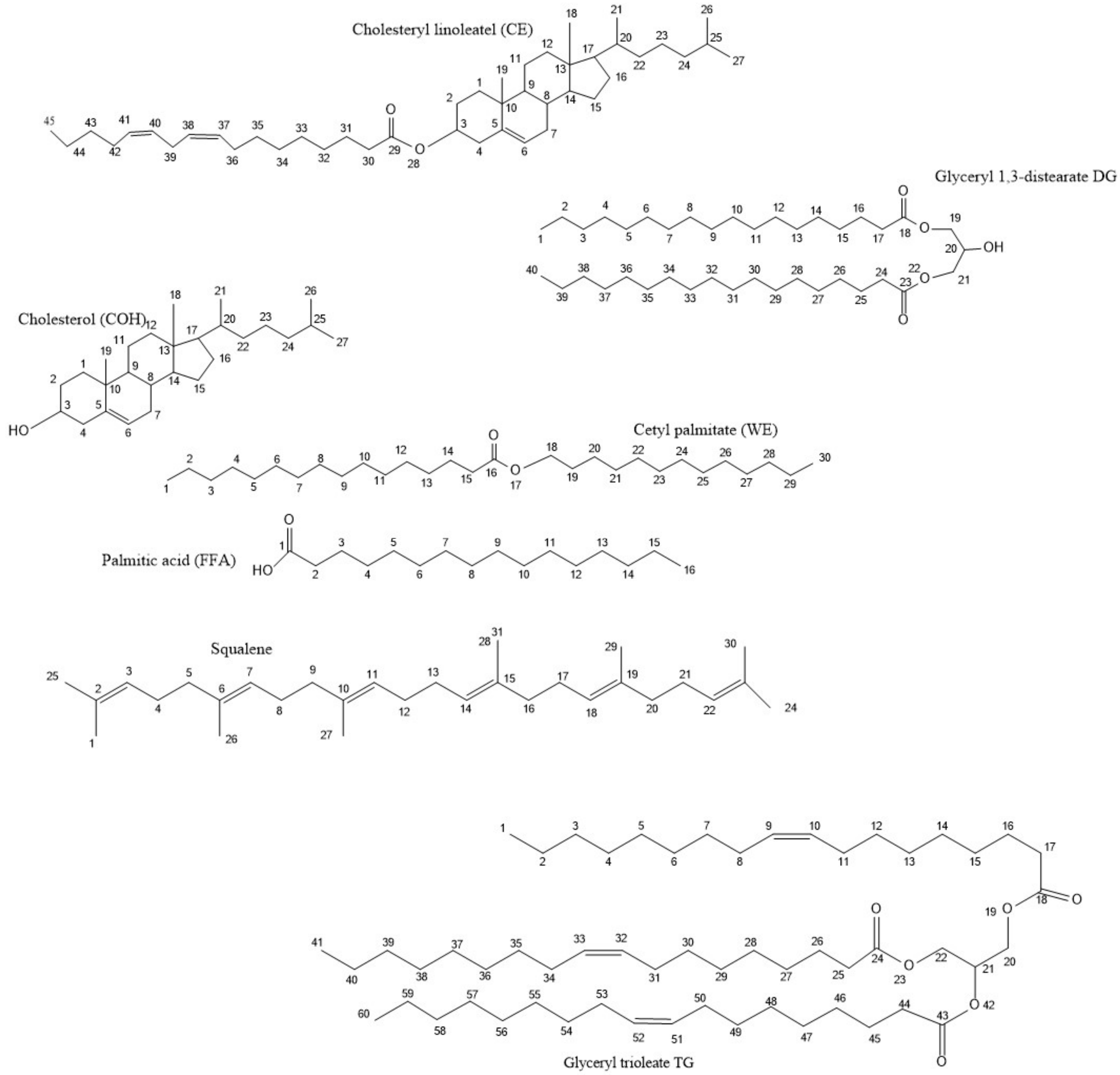

Figure S1 standard lipids molecules with numbering referring to proton assignment.

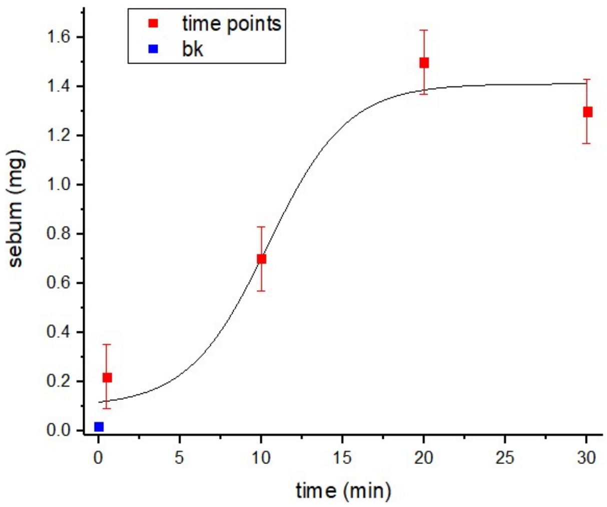

Figure S2. ^{1}H-NMR determined weight of sebum obtained at different Sebutape® application time collected from healthy volunteer. In blue average value obtained from clean tapes (blank).
